# MMP1 and MMP9 are potential prognostic biomarkers and targets for uveal melanoma

**DOI:** 10.1186/s12885-021-08788-3

**Published:** 2021-09-29

**Authors:** Tianyu Wang, Yuanyuan Zhang, Jianhao Bai, Yawen Xue, Qing Peng

**Affiliations:** Department of Ophthalmology, Shanghai Tenth People’s Hospital, Tongji University School of Medicine, Shanghai, People’s Republic of China

**Keywords:** Matrix metallopeptidase, Transcriptional level, Protein-protein interaction, Prognosis, Overall survival, Disease-free survival

## Abstract

**Background:**

Uveal melanoma (UVM) is the leading cause of eye-related mortality worldwide. This study aimed to explore the expression and prognostic value of matrix metalloproteinases (MMPs) in UVM.

**Methods:**

Gene expression levels were obtained from the Gene Expression Omnibus (GEO) and Oncomine databases. Functional and pathway enrichment analyses were performed using the Metascape database. GeneMANIA was then applied to construct a protein-protein interaction network and identify the hub genes. Moreover, overall survival (OS) and disease-free survival (DFS) analysis for the hub genes was performed using the UALCAN and Gene Expression Profiling Interactive Analysis (GEPIA) online tool. Furthermore, TRRUST was used to predict the targets of the MMPs.

**Results:**

Our results revealed that the transcriptional levels of *MMP1, MMP9, MMP10, MMP11, MMP13, MMP14*, and *MMP17* were upregulated in UVM tissues compared to normal tissues. A protein-protein interaction (PPI) network was constructed and the top 50 hub genes were identified. The functions of MMPs and their neighboring proteins are mainly associated with ECM-receptor interaction, proteoglycans in cancer, the IL-17 signaling pathway, and microRNAs in cancer. Among the MMPs, MMP1/2/9/11/14/15/16/17/24 played significant roles in the progression of UVM from stage 3 to stage 4. We also found that the expression of *MMP1, MMP2, MMP9, and MMP16* positively correlated with OS and DFS in patients with UVM*.* Additionally, 18 transcription factors associated with nine MMPs were identified.

**Conclusions:**

The results of this study may provide potential biomarkers and targets for UVM. However, further studies are required to confirm these results.

## Background

Uveal melanoma (UVM) is the most common primary malignant tumor of the eye in adults [1]. It often occurs in people aged 50–60 years. The clinical manifestations, complications, high degree of malignancy, and poor prognosis of UVM seriously threaten the visual function and life of patients [2]. At present, the main treatment methods for UVM are enucleation, photocoagulation, local tumor resection, and external scleral application radiotherapy [1, 3]. Although these treatments have achieved a certain degree of success, there are still some problems. The most common sites of metastasis are the liver, lung, and bone. Furthermore, the mortality rate of UVM is as high as 50% [4]. Currently, the etiology and molecular mechanisms of UVM are still unclear [5, 6]. The abnormal expression of multiple genes may be involved. Therefore, finding new molecular therapeutic targets to establish early diagnosis and inhibit tumor metastasis is key to improving the survival rate of patients.

To date, 26 MMPs have been investigated, most of which are present in the human proteome [7]. MMPs can degrade various protein components in the extracellular matrix. Meanwhile, they can also destroy the histological barrier of tumor cell and affect tumor migration, invasion, metastasis, and angiogenesis [8–12]. Therefore, MMPs are considered as the main proteolytic enzymes in this process and the relation between MMPs and extracellular matrix components is interactive. According to their substrates, MMPs can be divided into six categories: (1) collagenases, including MMP1 (collagenase-1), MMP8 (collagenase-2), MMP13 (collagenase-3) with the main function of degrading collagen I, II, and III; (2) gelatinases, including MMP2 (gelatinase A) and MMP9 (gelatinase B), with the function of degrading collagen I and IV; (3) stromelysins, including MMP3 (stromelysin-1) and MMP11 (stromelysin-3); (4) MMP7. Its substrates are elastin, fibronectin, and laminin. They are necessary for the epithelization of mucosal wounds; (5) MMP12, the substrates of MMP12 are mainly type IV collagen, gelatin, fibronectin, and laminin; (6) Membrane-type MMPs (MT-MMPs), including MMP14, MMP15, MMP16, MMP17, MMP24, and MMP25. The substrates of MMP14 are type I, II, and III collagen. They can promote keratinocyte growth, airway re-epithelization, and cell migration. However, the role of the other MT-MMPs remains unclear. MMP activity is regulated at three levels: gene transcription, the activation of enzyme precursors and the effect of tissue inhibitors of metalloproteinases (TIMP).

In recent years, studies have shown that MMPs expression is associated with the occurrence and development of a variety of tumors. These molecules are important in the growth and invasion of malignant tumors and their expression has an important impact on the prognosis of patients [13–16] . However, there are few reports on the MMPs gene family role in the prognosis of UVM.

In recent years, with the development of high-throughput technology, gene chips and gene sequencing have become necessary and efficient methods for studying tumor diseases. In the era of big data, much genomic, epigenomic, and transcriptomic data are available in online databases. However, effective data mining remains lacking. This study used bioinformatic methods to detect the expression and mutation of the core MMPs, aiming to screen the genes closely related to the occurrence, development, and prognosis of UVM through clinical big data. We aimed to provide a reference for the clinical treatment of UVM, by establishing a theoretical basis for the research and development of targeted therapies for this malignancy.

## Methods

Since all the data were retrieved from the online databases, we assumed that all written informed consent had been already obtained.

### Malacards database

MalaCards (https://www.malacards.org) is an integrated database of human maladies and their annotations, modeled on the architecture and richness of the popular GeneCards database of human genes [17]. In this study, we aimed to identify more potential classical genes in UVM.

### Oncomine database

ONCOMINE (www.oncomine.org) is a classic cancer sample database. It is a cancer microarray database and integrated data mining platform which aims to promote the discovery of genome-wide expression analysis [18, 19]. In this study, transcriptional differential expression of 17 different MMPs between different cancer tissues and their corresponding adjacent normal controls were obtained from the ONCOMINE database. Student’s t-test was used to compare the differences in transcriptional expression. Fold change and cut-off of *p*-value were as follows: fold change, 1.5; *p* value, 0.01; gene rank, 10%; data type, mRNA.

### TCGA data and cBioPortal

TCGA (https://www.cancer.gov/about-nci/organization/ccg/research/structural-genomics/tcga) is currently the largest cancer gene information database that is comprehensive in many cancer types and multi-omics data [20]. We selected a UVM dataset (TCGA, Firehose Legacy) including 80 samples and further analyzed the expression of MMPs using cBioPortal (http://www.cbioportal.org/) [21]. Mutations, putative copy-number alterations from GISTIC, and mRNA expression z-scores relative to diploid samples (RNA Seq V2 RSEM) were included in the genomic map.

### Metascape

Metascape (http://metascape.org/gp/index.html#/main/step1) is a public online analysis database used to perform Gene Ontology (GO) and Kyoto Encyclopedia of Genes and Genomes (KEGG) enrichment analyses [22]. In our study, Metascape was used for functional annotation and enrichment analysis of MMPs and their 50 neighboring genes and the relevant parameters were as follows: minimum overlap, 3; *p* value cutoff, 0.01; minimum enrichment, 1.5.

### GeneMANIA

GeneMANIA (http://genemania.org/) is an online prediction website that provides information on protein-DNA, protein-protein, and genetic interactions, reactions, pathways, and protein domains of related genes [23]. In our study, GeneMANIA was used to compare the correlation between MMPs in UVM patients.

### GEPIA 2

Based on tumor and normal samples from the TCGA and GTEx databases, GEPIA 2 (http://gepia2.cancer-pku.cn/#index) is an indispensable and highly cited database for gene expression analysis [24]. It can be used to perform a survival analysis of specific genes. In GEPIA 2, UVM patients were divided into low and high expression groups based on the median values of mRNA expression and validated by GEPIA 2 survival curves. The methods used were OS and DFS with a cut-off of 50%.

### UALCAN

UALCAN (http://ualcan.path.uab.edu/index.html) is an effective website for online analysis and mining of cancer data, mainly based on the relevant cancer data in the TCGA database [25]. In this study, UALCAN was used to analyze the mRNA expression of 17 different MMP family members in primary UVM tissues and its association with clinicopathological parameters. Student’s t-test was used to compare the difference in transcriptional expressions and statistical significance was set at *p* < 0.05.

### TRRUST

TRRUST (https://www.grnpedia.org/trrust/) is a curated database of human and mouse transcriptional regulatory networks that contains information derived from 11,237 published articles [26]. In our study, this database was used to explore the association between MMPs and transcriptional factors.

### Timer

TIMER (http://timer.cistrome.org/) is a comprehensive resource for systematical analysis of immune infiltrations across diverse cancers [27–29]. “Immune association” and “gene” module were used in this study to evaluate the correlation between MMPs and the infiltration of immune cells. R software and GSVA package were also used to draw the pictures of associations between MMPs and the immune cells [30, 31].

## Results

### Eight elite genes related to UVM

Using MalaCards, we identified eight elite genes related to UVM. The relationship between these elite genes and UVM comes from artificial annotation and reliable resources (Table 1).

**Table 1:** 8 elite genes related to uveal melanoma

Number	Gene	Description	Category	Score
1	BAP1	BRCA1 Associated Protein 1	Protein coding	764.58
2	GNAQ	G Protein Subunit Alpha Q	Protein coding	759.14
3	GNA11	G Protein Subunit Alpha 11	Protein coding	759.09
4	PLCB4	Phospholipase C Beta 4	Protein coding	407.24
5	CYSLTR2	Cysteinyl Leukotriene Receptor 2	Protein coding	356.9
6	UVM1	Melanoma, Uveal, Susceptibility To, 1	Genetic Locus	57.97
7	UVM2	Melanoma, Uveal, Susceptibility To, 2	Genetic Locus	57.97
8	SF3B1	Splicing Factor 3b Subunit 1	Protein coding	38.14

### MMPs transcriptional differences in melanoma cancers and normal tissues

**Fig. 1:**
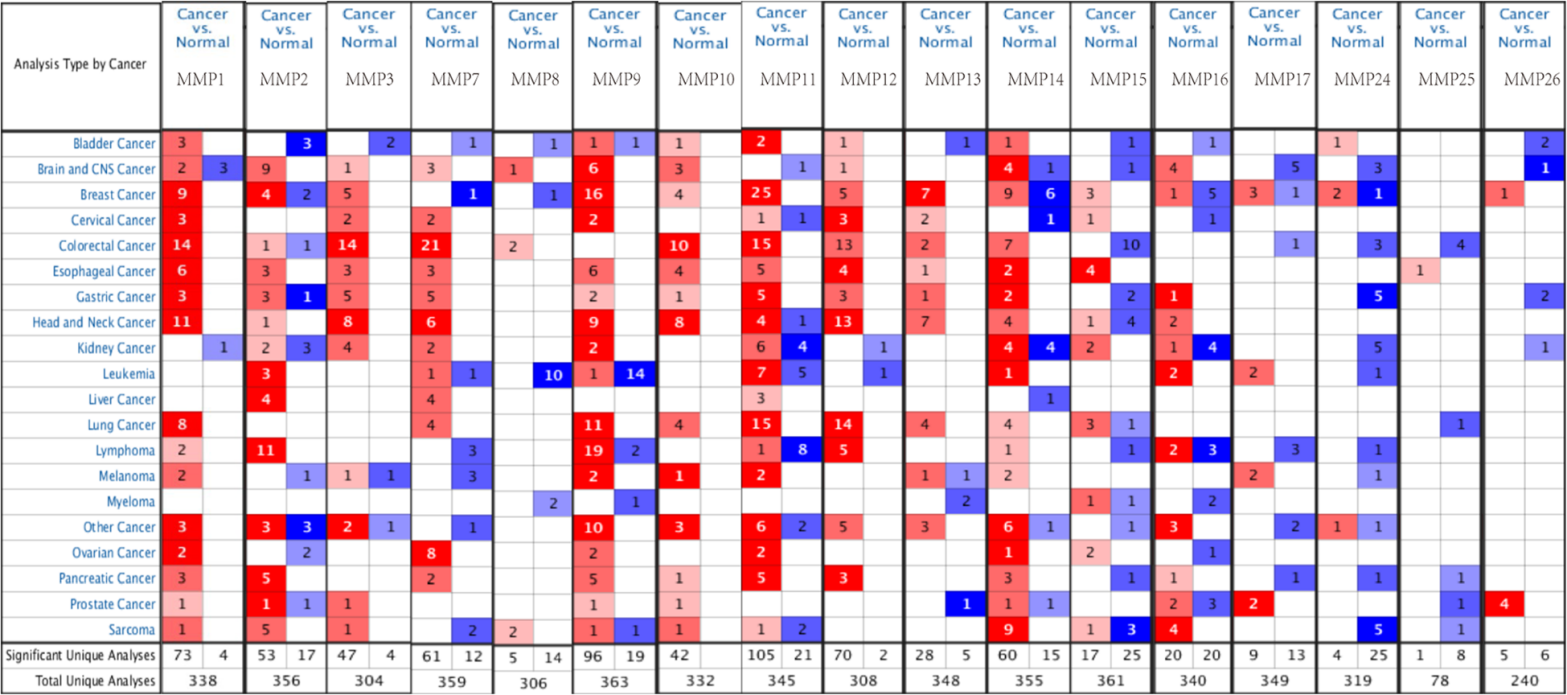
Transcriptional expression of MMPs in 20 different types of cancer diseases (ONCOMINE database). Difference of transcriptional expression was compared by students’ t-test. Cut-off of *p* value and fold change were as following: *p* value: 0.01, fold change: 1.5, gene rank: 10%, data type: mRNA

We used the ONCOMINE database to explore the expression levels of MMPs in UVM and normal tissues (Fig. 1). This analysis revealed that the transcriptional levels of *MMP1, MMP9, MMP10, MMP11, MMP13, MMP14*, and *MMP17* were upregulated in patients with UVM, while the transcriptional levels of *MMP2, MMP7*, and *MMP24* were lower than those in normal samples. Next, we discuss the relationship between UVM and *MMP1, MMP2, MMP9, MMP11, MMP14, MMP15, MMP16, MMP17*, and *MMP24*.

### GO and KEGG explorations of MMPs in UVM patients

Through GeneMANIA analysis, we identified 50 neighboring genes with the highest frequency association with MMPs. These data indicated that *CTB-96E2.2*, *HPX*, *PRG4*, *VTN*, *ASTL*, *MEP1B*, *MFAP2*, *MEP1A*, *BSPH1*, *ELSPBP1*, *IGF2R*, *ENDOU*, *TLL2*, *TLL1*, *BMP1*, *SEL1L*, *FN1*, *MFAP5*, *F12*, *MRC2, LY75-CD302*, *LY75*, *TIMP2*, *HGFAC*, *MRC1*, *TINAG*, *PLA2R1*, *ENPP2*, *ENPP1*, *IGFBP1*, *TIMP1*, *TFPI*, *OSCAR*, *SPP1*, *TIMP4*, *ENPP3*, *PTAFR*, *TIMP3*, *PIK3IP1*, *LAIR1*, *SUSD2*, *GP6*, *TARM1*, *FCAR*, *CRISP3*, *CRISP2*, *PROC*, *SDC1*, *VSTM1*, and *CRISP1* were associated with the function and pathway of MMPs in patients with UVM. The functions of MMP members and their neighboring genes were predicted using Metascape.

**Fig. 2:**
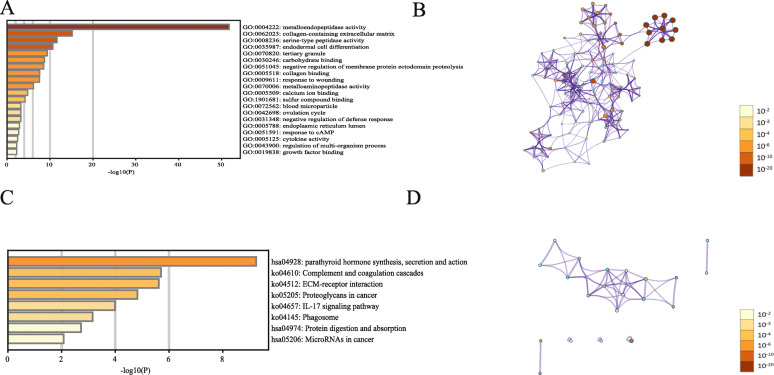
PPI and functional enrichment analysis of MMPs in patients with UVM (Metascape). **a** MMPs gene ontology (GO) enriched terms, colored by *p*-value. **b** Networks of GO enriched terms colored by *p* value, and terms containing more genes tend to have a more significant *p* value. **c** MMPs Kyoto Encyclopedia of Genes and Genomes (KEGG) enrichment terms, colored by *p*-value. **d** Networks of KEGG enriched terms colored by *p* value, and terms containing more genes tend to have a more significant *p* value

The biological process (BP) functions for these genes were primarily endodermal cell differentiation, negative regulation of membrane protein ectodomain proteolysis, response to wounding, ovulation cycle, negative regulation of defense response, response to cAMP, and regulation of multi-organism processes. The cellular components (CC) related to these genes were mainly collagen-containing extracellular matrix, tertiary granules, blood microparticles, and endoplasmic reticulum lumen. The identified molecular functions (MF) related to these genes were metalloendopeptidase activity, serine-type peptidase activity, carbohydrate binding, collagen binding, metalloaminopeptidase activity, calcium ion binding, sulfur compound binding, cytokine activity, and growth factor binding (Fig. 2A, B, Table 2).

**Table 2:** The GO function enrichment analysis of MMP family members and neighboring genes (Metascape)

GO	Category	Description	Count	%	Log10(P)	Log10(q)
GO:0004222	GO Molecular Functions	metalloendopeptidase activity	29	39.73	−51.76	−47.41
GO:0062023	GO Cellular Components	collagen-containing extracellular matrix	17	23.29	−15.20	−11.96
GO:0008236	GO Molecular Functions	serine-type peptidase activity	11	15.07	−11.66	−8.46
GO:0035987	GO Biological Processes	endodermal cell differentiation	7	9.59	−10.68	−7.54
GO:0070820	GO Cellular Components	tertiary granule	9	12.33	−9.36	−6.33
GO:0030246	GO Molecular Functions	carbohydrate binding	10	13.70	−8.74	−5.77
GO:0051045	GO Biological Processes	negative regulation of membrane protein ectodomain proteolysis	4	5.48	−8.54	−5.59
GO:0005518	GO Molecular Functions	collagen binding	6	8.22	−7.62	−4.78
GO:0009611	GO Biological Processes	response to wounding	13	17.81	−7.55	−4.73
GO:0070006	GO Molecular Functions	metalloaminopeptidase activity	4	5.48	−6.16	−3.57
GO:0005509	GO Molecular Functions	calcium ion binding	10	13.70	−4.82	−2.42
GO:1901681	GO Molecular Functions	sulfur compound binding	6	8.22	−4.22	−1.89
GO:0072562	GO Cellular Components	blood microparticle	4	5.48	−3.24	−1.00
GO:0042698	GO Biological Processes	ovulation cycle	3	4.11	−3.23	−1.00
GO:0031348	GO Biological Processes	negative regulation of defense response	5	6.85	−3.22	−0.99
GO:0005788	GO Cellular Components	endoplasmic reticulum lumen	5	6.85	−2.92	−0.72
GO:0051591	GO Biological Processes	response to cAMP	3	4.11	−2.72	−0.56
GO:0005125	GO Molecular Functions	cytokine activity	4	5.48	−2.48	−0.34
GO:0043900	GO Biological Processes	regulation of multi-organism process	5	6.85	−2.25	−0.12
GO:0019838	GO Molecular Functions	growth factor binding	3	4.11	−2.25	−0.12

The top eight KEGG pathways for MMP family members and their neighboring genes are shown in Fig. 2C and Table 3. Among these pathways, ECM-receptor interaction, proteoglycans in cancer, IL-17 signaling pathway, and microRNAs in cancer were found to be related to carcinoma pathogenesis and metastasis (Fig. 2C, D, Table 3).

**Table 3:** The KEGG function enrichment analysis of MMP family and neighboring genes (Metascape)

GO	Category	Description	Count	%	Log10(P)	Log10(q)
hsa04928	KEGG Pathway	parathyroid hormone synthesis, secretion and action	8	10.96	−9.24	−6.34
ko04610	KEGG Pathway	Complement and coagulation cascades	5	6.85	−5.70	−3.21
ko04512	KEGG Pathway	ECM-receptor interaction	5	6.85	−5.62	−3.21
ko05205	KEGG Pathway	Proteoglycans in cancer	6	8.22	−4.83	−2.70
ko04657	KEGG Pathway	IL-17 signaling pathway	4	5.48	−3.99	−2.00
ko04145	KEGG Pathway	Phagosome	4	5.48	−3.15	−1.36
hsa04974	KEGG Pathway	Protein digestion and absorption	3	4.11	−2.72	−0.99
hsa05206	KEGG Pathway	MicroRNAs in cancer	4	5.48	−2.08	−0.49

### Genetic alteration, co-expression, and interaction analyses of MMPs in patients with UVM

**Fig. 3:**
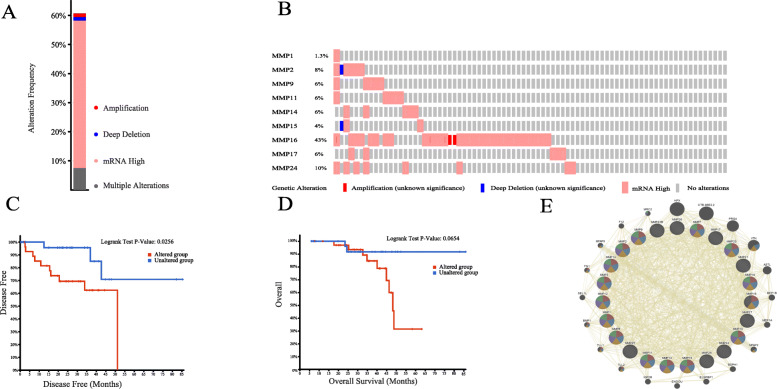
Alteration frequency of MMPs family members and neighboring gene network in patients with UVM (cBioPortal and geneMANIA). **a** Summary of alterations in 9 MMPs family members. **b** OncoPrint visual summary of alteration on a query of MMPs family members. **c** Kaplan-Meier plots comparing OS in patients with/without MMPs family member gene alterations. **d** Kaplan-Meier plots comparing DFS in patients with/without MMPs family member gene alterations. **e** Gene-gene interaction network of different expressed MMPs. Each node represents a gene, and the size of which represents the strength of the interaction

The cBioportal online tool was used to analyze the alteration frequency of MMP mutations in patients with UVM. According to the TCGA dataset, among the 80 UVM samples, MMPs were changed in 49 samples (61%) (Fig. 3A). *MMP16* (43%) had the highest genetic variation rate, while *MMP1* (1.3%) had the lowest. *MMP2, MMP9, MMP11, MMP14, MMP15, MMP17*, and *MMP24* were altered in 8, 6, 6, 6, 4, 6, and 10% of the patients with UVM, respectively (Fig. 3B). The result of Kaplan-Meier plotter and log-rank test showed no significant difference in OS between the altered and unaltered groups (log-rank test *p*-value =0.0654), while there was a significant difference in DFS (log-rank test p-value =0.0256) (Fig. 3C, D).

Moreover, a gene-gene interaction (GGI) network of 23 MMPs was constructed and analyzed using the GeneMANIA database (Fig. 3E). Twenty genes surrounding the 17 MMPs were significantly associated with each other in the shared protein, prediction, co-expression, pathway, and co-localization parameters. The top five genes most associated with MMPs were *HPX* (hemopexin), *CTB-96E2.2*, *PRG4* (proteoglycan 4), *VTN* (vitronectin), and *ASTL* (astacin-like metalloendopeptidase). Figure 3E shows that 95.59% of these genes had shared protein domains, 2.89% shared prediction, 0.72% shared physical interactions, and only 0.38% of these genes shared co-localization. Further functional analysis indicated that the most significant correlation was with the extracellular matrix (FDR = 2.85e-27). In addition, these genes were also correlated with extracellular structure, extracellular structure organization, collagen catabolic process, and multicellular organismal catabolic process.

### Correlation between MMPs and clinicopathological parameters in patients with UVM

**Fig. 4:**
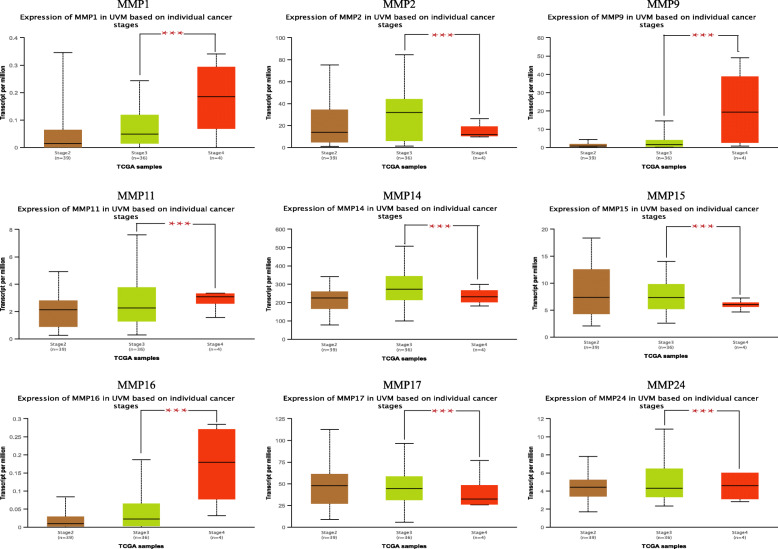
Correlation between 9 MMPs expression and tumor stage in UVM patients (GEPIA). The *p* value was set at 0.05. *** represents *p* < 0.001

The correlation between MMPs and the pathological and histological subtypes of UVM was further assessed by UALCAN (Fig. 4). As shown in Fig. 4, for patients with UVM, all MMPs above were markedly statistically significant during the transition from stage 3 to stage 4. These data strongly suggested that these MMPs play significant roles in the progression from stage 3 to stage 4.

### Prognostic value of mRNA expression of MMPs in patients with UVM

**Fig. 5:**
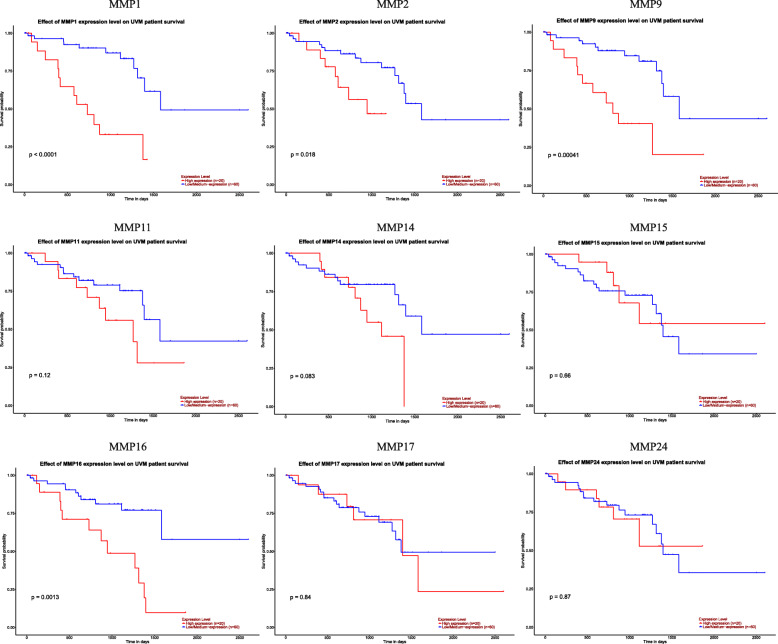
The prognostic value of 9 MMPs family members in UVM patients (UALCAN). The OS survival curves comparing patients with high (red) and low (blue) MMPs family member expressions were plotted. The threshold of *p* value is 0.05

In addition, we also obtained survival plots of MMPs in the uveal melanoma TCGA dataset from UALCAN. Kaplan Meier plots showed the effect of MMPs on patients’ OS (Fig. 5). The mRNA expression of four MMPs was significantly associated with the prognosis of UVM. The increased mRNA levels of *MMP1, MMP2, MMP9, and MMP16* were strongly associated with poor OS, while those of *MMP14, MMP15, MMP17, and MMP24* were not related to OS in UVM.

**Fig. 6:**
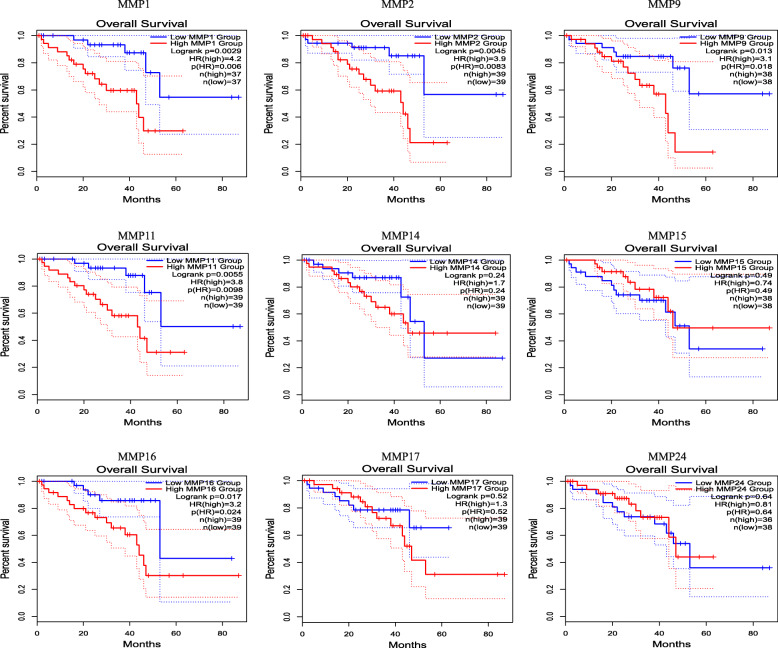
The prognostic value of 9 MMPs family members in UVM patients (GEPIA). The DFS survival curves comparing patients with high (red) and low (blue) MMPs family member expressions were plotted. The threshold of *p* value is 0.05

**Fig. 7:**
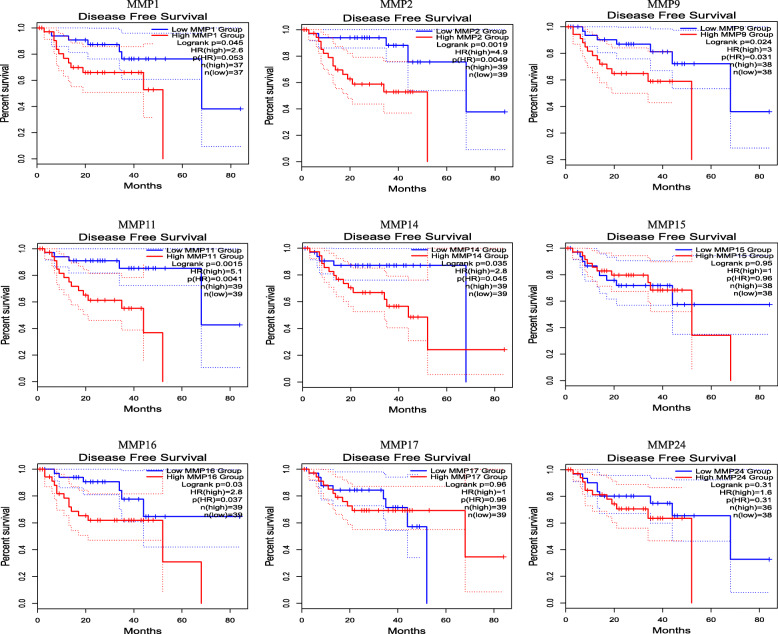
The prognostic value of 9 MMPs family members in UVM patients (GEPIA). The OS survival curves comparing patients with high (red) and low (blue) MMPs family member expressions were plotted. The threshold of *p* value is 0.05

We also used the GEPIA dataset to compare the mRNA expression of MMPs to the OS and DFS of patients with UVM. DFS and OS curves are presented in Figs. 6 and 7. There were significant associations between some *MMPs* (*MMP1, MMP2, MMP9, MMP11*, and *MMP16*) and OS in patients, which was almost consistent with the results presented above. Moreover, there were significant associations between certain *MMPs* (*MMP1, MMP2, MMP9, MMP11, MMP14,* and *MMP16*) and DFS.

Taken together, these results showed that *MMP1, MMP2, MMP9* and *MMP16* expression levels may be exploited as potential biomarkers for the prediction of the survival of patients with UVM.

### Transcription factor targets in patients with UVM

Owing to the significant difference in the expression of MMPs in UVM vs. normal tissues, we explored related transcription factor targets of the differentially expressed MMPs using TRRUST DATABASE. *MMP1, MMP2, MMP9, MMP11, MMP14, MMP15, MMP16, MMP17,* and *MMP24* were included in TRRUST. We found that 18 transcription factors (*MAZ, ETV4, SRF, ETS2, STAT3, KLF8, RELA, NFKB1, SNAI2, NFKBIA, JUN, SP1, TWIST1, FOS, PPARG, TFAP2A, ETS1*, and *TP53*) were associated with the regulation of MMPs (Table 4).

**Table 4:** Transcription factors of the differentially expressed MMPs

Key TF	Description	*P* value	FDR	Regulated genes
MAZ	MYC-associated zinc finger protein (purine-binding transcription factor)	4.20E-09	7.55E-08	MMP9, MMP14, MMP1
ETV4	ets variant 4	1.15E-07	1.03E-06	MMP14, MMP1, MMP2
SRF	serum response factor (c-fos serum response element-binding transcription factor)	1.72E-07	1.03E-06	MMP14, MMP2, MMP9
ETS2	v-ets erythroblastosis virus E26 oncogene homolog 2 (avian)	3.69E-07	1.35E-06	MMP1, MMP9, MMP2
STAT3	signal transducer and activator of transcription 3 (acute-phase response factor)	3.76E-07	1.35E-06	MMP9, MMP2, MMP1, MMP14
KLF8	Kruppel-like factor 8	5.65E-06	1.70E-05	MMP14, MMP9
RELA	v-rel reticuloendotheliosis viral oncogene homolog A (avian)	7.50E-06	1.73E-05	MMP9, MMP2, MMP14, MMP1
NFKB1	nuclear factor of kappa light polypeptide gene enhancer in B-cells 1	7.70E-06	1.73E-05	MMP1, MMP2, MMP9, MMP14
SNAI2	snail homolog 2 (Drosophila)	1.83E-05	3.67E-05	MMP17, MMP9
NFKBIA	nuclear factor of kappa light polypeptide gene enhancer in B-cells inhibitor, alpha	3.44E-05	6.20E-05	MMP1, MMP9
JUN	jun proto-oncogene	3.91E-05	6.40E-05	MMP9, MMP2, MMP1
SP1	Sp1 transcription factor	4.40E-05	6.61E-05	MMP2, MMP9, MMP11, MMP14
TWIST1	twist basic helix-loop-helix transcription factor 1	0.000119	0.000165	MMP2, MMP1
FOS	FBJ murine osteosarcoma viral oncogene homolog	0.000318	0.000409	MMP9, MMP1
PPARG	peroxisome proliferator-activated receptor gamma	0.000427	0.000512	MMP9, MMP1
TFAP2A	transcription factor AP-2 alpha (activating enhancer binding protein 2 alpha)	0.000494	0.000556	MMP2, MMP9
ETS1	v-ets erythroblastosis virus E26 oncogene homolog 1 (avian)	0.000611	0.000647	MMP1, MMP9
TP53	tumor protein p53	0.0026	0.0026	MMP1, MMP2

### Immune cell infiltration of MMPs in patients with UVM

**Fig. 8:**
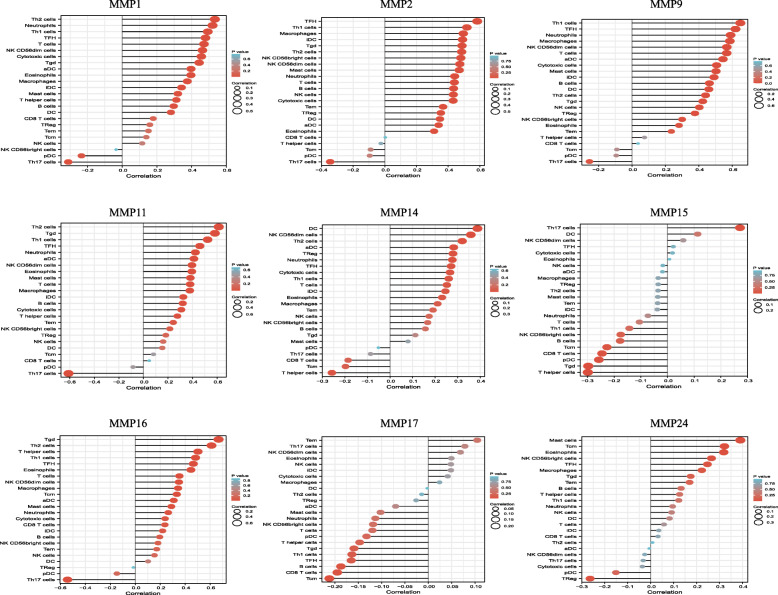
The relationship between differentially expressed MMPs and immune cell infiltration (TIMER)

MMPs are involved in cancer-related inflammation and immune cell infiltration, thus affecting the clinical outcome of UVM patients. Therefore, the TIMER database was used to evaluate the comprehensive relationship between differentially expressed MMPs and immune cell infiltration. The results are presented in Fig. 8. Except for MMP15 and MMP17, seven matrix metalloproteinases (including MMP1, MMP2, MMP9, MMP11, MMP14, MMP16, MMP24) positively correlated with the infiltration of most of the immune cells. We also found that MMP15 and MMP17 negatively correlated with the infiltration of most of the immune cells.

## Discussion

Numerous studies have suggested that dysregulation of MMPs is involved in the development and progression of many tumors. Although some members of MMPs have been shown to play crucial roles in carcinoma, the distinct roles of MMP family members in UVM remain to be elucidated. In this study, the mutations, mRNA expression, prognostic values, and potential functions of different MMP family members in UVM were identified with bioinformatics tools.

MMP proteins are associated with the breakdown of the extracellular matrix during physiological processes and disease processes. Mutations in *MMP1* are associated with chronic obstructive pulmonary disease [32]. Moreover, recent studies have shown that *MMP1* is highly expressed in human oral squamous cell carcinoma and cervical squamous cell carcinoma. It can promote tumor growth and cell motility, participate in the development of human oral squamous cell carcinoma [33], and serve as a potential biomarker for cervical squamous cell carcinoma [12]. In our study, *MMP1* was found to be upregulated in UVM tissues, and high expression indicated shorter OS and DFS, which was consistent with the results of previous studies.

MMP2 is a zinc-dependent enzyme that cleaves the components of the extracellular matrix and molecules. Unlike most MMP family members, activation of MMP2 occurs on the cell membrane. This enzyme can be activated extracellularly and intracellularly and is involved in multiple pathways. Mutations in this gene are associated with the Winchester syndrome [34]. Recent studies have indicated that *MMP2* is highly expressed in many tumors, and its increase promotes proliferation and metastasis, and reduces tumor cell apoptosis [35–38]. In our study, *MMP2* was found to be upregulated in UVM, and high expression indicated shorter OS and DFS.

Like other MMP family members, *MMP9* is involved in disease processes such as metastasis and arthritis, as well as in the degradation of extracellular matrix components in normal processes such as reproduction and tissue remodeling. MMP9 has been found to play an essential role in cancer progression and is significantly associated with metastasis, angiogenesis, and tumor growth [39]. Dysregulation of MMP9 is involved in various diseases, such as neurological disorders [40], inflammatory diseases [41], cardiovascular diseases [16], and lung diseases [8]. In our study, *MMP9* was found to be upregulated in UVM, and high expression indicated shorter OS and DFS, which was consistent with the results of previous studies.

*MMP11* is a protein-coding gene, and *MMP25* is an important paralog of this gene. MMP11, a zinc-dependent endopeptidase and the main protease involved in ECM degradation, plays an important role in various biological processes, such as breast cancer [13], prostate cancer [9], gastric cancer [42], and lung cancer [43], where the high expression of *MMP11* is associated with metastasis and a poor clinical outcome. Furthermore, several studies have shown that *MMP11* is a potential biomarker gene in breast cancer, which may facilitate the diagnosis and prognosis of breast cancer [44, 45]. In our study, *MMP11* was found to be upregulated in UVM, and high expression indicated shorter OS and DFS, which was consistent with the results of previous studies.

The protein encoded by *MMP14* is a member of the membrane-type MMP (MT-MMP), which contains a potential transmembrane domain, indicating that it is expressed on the cellular surface.

Studies have reported that high *MMP14* expression is associated with poor prognosis and might serve as a marker of progression in muscle-invasive bladder cancer and colorectal cancer [10, 46]. Low *MMP14* expression can inhibit the invasion and metastasis of breast cancer and gastric cancer cells [47, 48]. In our study, *MMP14* was found to be upregulated in UVM, and high expression indicated shorter DFS, but it was not associated with OS in patients with UVM.

Like *MMP14,* the protein encoded by *MMP15* is also a member of the MT-MMP. MMP15, also known as MT2-MMP, was first extracted from a human lung cDNA library.

It is composed of 669 amino acids encoded in 3530 bp of the gene. Proteins in this family are associated with the breakdown of the extracellular matrix in various diseases. *MMP15* is highly expressed in bladder cancer and contributes to inflammation and angiogenesis in cancer cells [49]. MMP15 is also associated with malignancy, aggressiveness, and survival prognosis in human urinary bladder cancer by activating other MMPs [50]. The expression of *MMP15* is significantly higher in tumors with a higher level of aggressiveness and malignancy [15]. In our study, *MMP15* was not found to be upregulated in UVM and the expression of *MMP15* was not associated with OS or DFS in patients with UVM.

MMT16 was once referred to as MT-MMP2 and is has been renamed as MT-MMP3 or MMP16.

MMP16 is activated when cleaved by other proteinases. Degradation of extracellular and microRNAs in cancer is an important pathway related to MMP16. *MMP24* is an important paralog of the *MMP16* gene. Some studies have indicated that high expression of *MMP16* is associated with the proliferation, migration, and invasion of cutaneous squamous cell carcinoma cells [51], while knockdown of *MMP16* could inhibit cell proliferation and invasion in chordoma cells [11]. Moreover, some variations in *MMP16* have been found to be associated with different types of diseases [52, 53]. In our study, *MMP16* was found to be upregulated in UVM, and high expression was associated with shorter OS and DFS.

*MMP17* is a protein-coding gene that is associated with diseases such as breast cancer [14], colon cancer [54], and head and neck cancer [55]. While the expression of *MMP17* has been associated with different physiological and pathological processes, the mechanisms underlying these processes remain unknown. In our study, *MMP17* was not found to be upregulated in UVM and the expression of *MMP17* was not associated with OS and shorter DFS in UVM.

The MMP24 protein is associated with the breakdown of the extracellular matrix in disease processes and normal physiological processes. The proteins cadherin 2 and MMP2 are substrates of this protease. *MMP16* is an important paralog of this gene.

In the present study, we comprehensively assessed the expression and prognostic value of 26 MMPs in UVM. In our study, *MMP24* was not found to be upregulated in UVM and the expression of *MMP24* was not associated with overall survival and shorter disease-free survival in uveal melanoma patients. However, MMP24 play a significant role in the progression from stage 3 to stage 4 for UVM patients.

To date, our study is the first bioinformatic analysis to explore the association between UVM and all the members of the matrix metalloproteinase family. However, there are some limitations in our study. First, due to the limitation of conditions, there is no relevant experimental verification in our study. At the appropriate time in the future, we will carry out related experiments. Second, due to the lack of clinical data on UVM in relevant databases, such as TCGA, we could not find sufficient data for an effective analysis.

## Conclusions

In conclusion, the current study suggests that the transcription levels of *MMP1/9/10/11/13/14/17* are obviously upregulated in UVM and may play an important role in its occurrence and progression. *MMP 1/2/9/11/14/15/16/17/24* could be used as molecular markers to elucidate the stages of UVM. In addition, abnormal expression of *MMP1/2/9/11/16* was found to be related to OS in UVM, and *MMP1/2/9/11/14/16* were found to be associated with DFS. Moreover, the expression of *MMP1, MMP2, MMP9*, and *MMP16* was positively correlated with OS and DFS. We also found that most MMPs correlated with the infiltration of immune cells. Overall, our results revealed that *MMP1* and *MMP9* may serve as potential prognostic biomarkers and as therapeutic targets for UVM. However, further studies are needed to confirm our results and assess the clinical utility of MMPs.

## Data Availability

The datasets generated and/or analyzed during the current study are available in the Oncomine repository, [https://www.oncomine.org/resource/main.html]; the TCGA respository, [https://www.cancer.gov/about-nci/organization/ccg/research/structural-genomics/tcga]; the Metascape respository, [http://metascape.org/gp/index.html#/main/step1]; the GeneMANIA respository, [http://genemania.org/]; GEPIA 2 respository, [http://gepia2.cancer-pku.cn/#index]; the UALCAN respository, [http://ualcan.path.uab.edu/index.html]; the TRRUST respository, [https://www.grnpedia.org/trrust/]; the TIMER respository, [http://timer.cistrome.org/].

## Abbreviations

UVM: Uveal melanoma
MMP: Matrix metalloproteinases
GEO: Gene Expression Omnibus
OS: Overall survival
DFS: Disease-free survival
GEPIA: Gene Expression Profiling Interactive Analysis
PPI: Protein-protein interaction
BP: Biological process
CC: Cellular component
MF: Molecular function
GO: Gene ontology
KEGG: Kyoto encyclopedia of genes and genomes
GGI: Gene-gene interaction
TCGA: The cancer genome atlas
